# Engaging early career researchers in a global health research capacity-strengthening programme: a qualitative study

**DOI:** 10.1186/s12961-022-00949-5

**Published:** 2023-03-16

**Authors:** Claire Hawcroft, Evelina Rossi, Nerissa Tilouche, Ana Flavia d’Oliveira, Loraine J. Bacchus

**Affiliations:** 1 Bristol Medical School, University of Bristol, 5 Tyndall Ave, Bristol, BS8 1UD United Kingdom; 2 Department of Global Health and Development, London School of Hygiene and Tropical Medicine, Keppel Street, London, WC1E 7HT United Kingdom; 3 Department of Public Health, Environments and Society, London School of Hygiene and Tropical Medicine, Keppel Street, London, WC1E 7HT United Kingdom; 4 Faculty of Medicine, University of Sao Paulo, Av. Dr. Arnaldo, 455, São Paulo, SP 01246-903 Brazil

**Keywords:** Research capacity-strengthening, Capacity-building, Early career researcher, Global health research, Violence against women, Gender-based violence, Training, Funding, English language

## Abstract

**Background:**

Research capacity-strengthening is recognized as an important component of global health partnership working, and as such merits monitoring and evaluation. Early career researchers are often the recipients of research capacity-strengthening programmes, but there is limited literature regarding their experience.

**Methods:**

We conducted a qualitative study as part of an internal evaluation of the capacity-strengthening programme of the international HERA (HEalthcare Responding to violence and Abuse) research group. Semi-structured interviews were conducted with group members, and thematic analysis was undertaken.

**Results:**

Eighteen group members participated; nine of these were early career researchers, and nine were other research team members, including mid-career and senior researchers. Key themes were identified which related to their engagement with and experience of a research capacity-strengthening programme. We explored formal/planned elements of our programme: mentoring and supervision; training and other opportunities; funding and resources. Participants also discussed informal/unplanned elements which acted as important facilitators and/or barriers to engaging with research capacity-strengthening: English language; open relationships and communication; connection and disconnection; and diversity. The sustainability of the programme was also discussed.

**Conclusions:**

Our study gives voice to the early career researcher experience of engaging with a research capacity-strengthening programme in a global health group. We highlight some important elements that have informed adaptations to our programme and may be relevant for consideration by other global health research capacity-strengthening programmes. Our findings contribute to the growing literature and important discussions around research capacity-strengthening and how this relates to the future directions of global health partnership working.

## Background

There is growing recognition of the importance of research capacity-strengthening (RCS) within global health. There is no single definition of RCS, but ESSENCE on Health Research states that *“research capacity strengthening includes any effort to increase the ability of individuals and institutions to undertake high-quality research and to engage with the wider community of stakeholders”* [1]. Terminology varies and includes capacity strengthening, building or development [2]. A developing body of research has been published over the last decade [2], but the evidence base has been criticized as “confusing, controversial and poorly defined” [3, p. 2], and a robust implementation science around health RCS has not been established [2]. Historically, capacity-strengthening was often an implicit component of global health research programmes; it is now often embedded as a planned element [1]. RCS may be considered a programme output in itself, warranting monitoring and evaluation (M&E) [1, 4]. Good practice recommendations [1] and frameworks have been developed to support evaluation [5–7], and many programmes now have built-in M&E [8]. M&E can provide transparency and enable equity within the research group, lead to real-time adaptation and improvement and provide accountability to funders [1]. Evaluating capacity-strengthening is not without challenges; programmes are often complex, long-term and context-specific, with subjective or difficult-to-measure outcomes, no unified definition and most frameworks prioritizing quantitative over qualitative measurements [1, 2, 9].

Early career researchers (ECRs) are those in the early stages of their academic careers, and may include pre- or postdoctoral researchers. Their experience of RCS may present different opportunities and challenges to researchers at different career stages. This is a critical time for the establishment of a future successful academic career, as ECRs need to develop a broad range of academic competencies beyond scientific and technical knowledge. This includes writing skills (for publication and grant applications), engagement and dissemination (policy, academic networks, media, teaching, presentations) and self-development (team working skills, developing a career strategy) [10]. ECRs can benefit from RCS through individual funding routes such as scholarships or fellowships, or via mentorship and training embedded in collaborative research projects. One important outcome of successful RCS is the retention of ECRs in academic careers. Some ECRs must balance their research alongside competing demands, including clinical work [10], family or caring responsibilities [11]. Without adequate training, supervision or funding, some ECRs may prioritize teaching over research [12], leave academia [13] or seek opportunities abroad, thereby contributing to “brain drain” in low- and middle-income countries (LMICs) [14].

Existing studies looking at the RCS experience of ECRs mostly describe RCS programmes in Africa, using surveys and routinely collected M&E data to highlight programmatic lessons learned. We found four qualitative studies exploring the ECR experience of RCS [11, 12, 15, 16]. Studies identified similar challenges faced by ECRs in different programmes, including variable access to good-quality supervision and mentorship; limited exposure to relevant training in current research methods; lack of opportunity to attend conferences; weak academic environments and research networks; poorly defined career pathways; limited access to funding; problematic programme and grant management practices; and limited research infrastructure such as Internet connectivity [10–13, 15–20]. Providing ECRs with appropriate support may mitigate some of these challenges. Harle [17] identified key support needs for postdoctoral researchers in sub-Saharan Africa: opportunities for peer connection, support to publish their work, time to develop new research proposals, funding, opportunity to deliver supervision and a supportive institutional context. Researchers have also discussed the sustainability of RCS programmes. At the individual level, it is important to equip researchers with the capabilities, skills and networks to sustain themselves as scholars and researchers after their training programme, thus retaining their talents in academia [13]. Langhaug et al. [12] identified five key components to sustainable career tracts for mental health researchers in Africa: (i) research positions; (ii) research skills; (iii) funding; (iv) LMIC research commitment; and (v) advocacy. The focus of RCS programmes needs to shift towards ECRs but also engage with RCS at the institutional level [17]. Locally led initiatives should draw on existing regional capacities and develop south–south collaboration as well as mutually beneficial global collaborations [8, 13].

HEalthcare Responding to violence and Abuse (HERA) is a global health research group seeking to develop and evaluate violence against women interventions in healthcare systems in LMICs. The University of Bristol and London School of Hygiene & Tropical Medicine in the United Kingdom co-lead the group. Collaborating partner institutions located in LMICs are the University of São Paulo in Brazil, Kathmandu University in Nepal, An-Najah National University in Palestine and the University of Peradeniya in Sri Lanka. Project management, supervision and mentoring are shared across the HERA group. Whilst the United Kingdom's team has a role in project management at the group level, LMIC principal investigators (PIs) are responsible for leading their country research projects and teams. Mentoring and supervision are organized multilaterally through joint PhD supervision between the United Kingdom and partner countries, in addition to mentoring relationships. The majority of HERA ECRs are located in LMIC teams, with just one United Kingdom-based high-income country (HIC) ECR at the time of this study undertaking the role of RCS lead. ECRs in HERA have wide-ranging experience and roles; some work as research assistants or research coordinators, and others are undertaking formal training programmes such as PhDs. At the time of this study, HERA had three distance-learning PhD students from LMICs who were jointly funded by the National Institute for Health and Care Research (NIHR) and the University of Bristol. Four ECRs from LMICs were undertaking self-funded PhDs at their home institutions. Some ECRs are employed full time by HERA, whilst others combine HERA work with other research or clinical practice.

Despite the importance of ECRs as the next generation of academic leaders and their centrality to many RCS programmes, there is limited research exploring their experience of participating in RCS. We undertook a qualitative study to explore the ECR experience of RCS, facilitators and barriers to engaging with the HERA RCS programme and the sustainability of the programme. This in-depth exploration of the ECR experience enabled us to adapt our HERA RCS programme to better meet their needs.

## Methods

### RCS approach within HERA

RCS has been prioritized as a key element within HERA, with a United Kingdom-based RCS lead initially appointed to oversee and evaluate this work, as required by our funder. We aimed to identify and strengthen existing skills and expertise across the group. A baseline assessment was carried out by all country teams, including the United Kingdom, to identify the availability of local research infrastructure and existing expertise/development needs in research methodologies, management, supervision and policy. We held a capacity-strengthening session at our first annual workshop week, where participants discussed approaches to RCS, agreed on principles of M&E and cocreated shared group values. Shared group values were further refined at the second annual workshop and through an online ranking exercise; they were finalized after this study were undertaken. Final HERA shared group values were: mutual learning, knowledge sharing, acknowledge differences between countries (needs and strengths), fair opportunity, equity, transparency and respect. In order to promote and embed shared ownership of the RCS agenda, we continually sought contributions and gained consensus on plans within the group, providing feedback at monthly group meetings.

RCS within HERA was facilitated through various activities at the level of the individual, country team, group and wider research environment (Table 1).

**Table 1:** HERA capacity-strengthening activities by level

Individual	• Attendance in person at international research methods short courses (e.g. 1-week qualitative methods course at a United Kingdom university) and conferences • Attendance at in-country courses and conferences • Participation in online courses and masterclasses • Supervision of PhD students • Mentoring of LMIC and HIC researchers
Country	• In-country training delivered by senior HERA researchers. e.g. weekly educational meetings or courses • Training sessions delivered by local non-HERA activists and researchers • Mentoring/training of country teams in research methods and theory by external (United Kingdom and non-United Kingdom) senior researchers • English language and English academic writing training
Group	• Annual international group workshop: methodological training sessions and presentation of research and results from country research groups • Bimonthly ECR virtual peer support and education group
Research environment	• Delivery of presentations and training to local students, researchers or professionals • Engagement with local stakeholders, e.g. government officials and nongovernmental organizations • Attendance and presentation of research findings at national and international conferences • Dissemination of research findings through media • Collaboration with other global health research groups

### Study design

M&E was embedded at the start of the HERA research programme, with plans approved by the group. A mixed-methods evaluation aimed to evaluate the reach, context and acceptability of our approach to RCS using routinely collected data, surveys and semi-structured interviews. The data presented in this paper is from the semi-structured interviews.

### Sample

At the midpoint of the HERA programme, all 35 HERA group members were sent an email invitation with a participant information sheet. Those who expressed interest were contacted by an external research assistant (ER or NT) to arrange an interview; 18 interviews were conducted. Sociodemographic characteristics of interview participants are summarized in Table 2.

**Table 2:** Sociodemographic characteristics of participants

Characteristic	Category	No.	%
Age (years)	20–29	6	33
	30–39	5	28
	40–49	3	17
	50 and over	4	22
Gender	Female	15	83
	Male	3	17
	Other	0	0
Country team	Brazil	2	11
	Nepal	3	17
	Palestine	2	11
	Sri Lanka	4	22
	United Kingdom	7	39
LMIC/HIC location	LMIC	11	61
	HIC	7	39
Career level	Early -career researcher	9	50
	Mid-career researcher	4	22
	Senior researcher	4	22
	Other	1	6

### Data collection

Interviews were conducted using online video conferencing software between March and July 2020. Participants were offered the use of a translator. Informed consent was taken verbally. Participants were reminded that they could decline to answer any question or stop the interview at any point. Demographic data was collected from each participant. Interviews were digitally recorded, professionally transcribed, then cleaned and anonymized by the research team. Mean interview duration was 63 min 33 s (range 44 min and 05 s–91 min and 50 s).

The interview topic guide drew on existing RCS literature and findings from the ongoing mixed-methods RCS evaluation. The topic guide addressed perceptions and experience of RCS; RCS programme activities; mentoring and supervisory relationships; impact of RCS within participants’ country; global health research infrastructure and governance; research outputs; leadership and teamwork; RCS programme management; group-defined values; and programme sustainability. Further adaptations were made as pertinent topics arose during initial interviews, including the impact of the COVID-19 pandemic.

### Data analysis

Transcripts were uploaded into NVivo12 which was used to organize data. Coding was undertaken by three researchers (CH, ER and NT). The first three interviews were double coded, and codes were refined to create an initial coding framework. After the next set of interviews, codes were checked for consistency of application, and the coding framework further modified. A mixed deductive and inductive approach was taken to thematic analysis, with researchers seeking to explore pre-existing areas of interest, but also open to original ideas presented by participants [21]. Researchers discussed themes, defining and refining them as they were identified. Themes of relevance to this paper were organized into a framework [22] to enable visualization of data representation across different interviews, including shared or divergent views. LJB viewed anonymized data extracts to enable her to guide and supervise the analytic process. The anonymized framework was shared with AFO who provided input around interpretation of data.

### Ethics

Ethical approval for the study was granted by the University of Bristol Research Ethics Committee (reference 80222).

## Results

Thirty-five HERA group members were invited to participate, and interviews were undertaken with 18 respondents. Nine respondents were ECRs; other respondents were research team members, including mid-career and senior researchers. All ECR participants were from LMICs.

We describe below the ways in which participants understood and made sense of RCS within HERA and explore three main themes which relate to planned aspects of the RCS programme. We then present four unplanned aspects, which were identified as important by interview participants. Finally, we explore perspectives on the sustainability of our approach.

### Formal/planned elements

- Mentoring and supervision
- Training and other opportunities
- Funding and resources

### Informal/unplanned elements

- English language
- Open relationships and communication
- Connection and disconnection
- Diversity

### Understandings of RCS within HERA

There were varied understandings of RCS within HERA. Many focused on the development of scientific and technical knowledge and skills through either attendance at training and courses, or collaborative working.

*I think capacity-strengthening means we should improve our knowledge, skills and all the abilities through different aspects.* (P12, ECR, LMIC)

Participants felt able to share their opinion about RCS and request specific training. Discussing the process through which RCS occurs, participants reflected on the bidirectional potential for learning; it was recognized that knowledge transfer was not simply a north-to-south endeavor and that LMIC researchers also transferred their research knowledge and skills. One United Kingdom researcher felt that this was somewhat overlooked at the first annual workshop, with a *colonialist* assumption that the United Kingdom team should deliver most of the teaching. Following discussion and reflection, there was much greater involvement of LMIC team members in planning the agenda and delivering training and presentations at the second annual workshop; participants reflected on this positively. Some participants discussed how RCS had impacted their own personal development, and others described the strengthening of the research team that occurred through development of researcher knowledge and skills, group working, team management and resources.

Some participants discussed ethical considerations regarding how RCS relates to power and resource imbalance in global health research partnerships. These views were mostly articulated by mid/senior United Kingdom participants, as well as a small number of LMIC ECRs; this may reflect varying levels of critical engagement with this topic at different levels of seniority and across different country groups. Some participants talked about a political or moral motivation with RCS work seen as having the potential to address the ongoing imbalance between high-income countries (HICs) and LMICs in global health research.

*…to overcome some of the sort of ideological power imbalances and even epistemological imbalances that are in the relationships between researchers in the north and the south.* (P15, non-ECR, United Kingdom)

One example given was the progression of terminology from “capacity-building” to “capacity-strengthening” which was seen as acknowledging existing skills amongst LMIC researchers and aiming to redress the power imbalance between HICs and LMICs. One ECR cautioned that the RCS agenda may promote northern over southern models of academic achievement and that groups needed to be able to discuss this openly.

*I think there’s this fine line in capacity-building in a way that we benefit a lot from all of the opportunities it bring us but in another way it’s sort of trying to qualify us to be more Anglo-Saxon like… I don’t think we should give up on capacity-strengthening for that. I think that would be too simplistic. I think we have to find ways to consider all those things and to manage that and to be very frank and open about them so that we can address them better.* (P2, ECR, LMIC)

### Formal/planned elements of the RCS programme

#### Mentoring and supervision

ECRs had varied mentoring and supervision arrangements. All received supervision from their own country PI(s); some also received supervision, mentorship or methodological training from colleagues outside of their country. All participants spoke positively about supervision from their country PIs, which took different forms, including weekly or monthly team meetings, personal supervision or informal coworking. The nature of these relationships varied between hierarchical and formal, informal and friendly, or a mixture. Characteristics valued in these relationships included being *caring, approachable* and *friendly*. Some participants described their country PI(s) as *role models*. One participant explained how their country team leadership set an ethos of learning and development for their research group.

*…they are very, very concerned about us learning. I think that’s the focus of the group you know to keep us together and to ensure we are taking this opportunity to learn and to develop our careers.* (P13, ECR, LMIC)

LMIC participants who received mentorship or methodological training from United Kingdom team members were positive about this experience. One non-ECR from a LMIC described a *chain reaction* of capacity-strengthening in which they are able to pass on what they learned through being mentored to their own ECR mentees.

*…after I get that guidance and way forward from them then you know I get to practice that same thing with my other colleagues. So I think it’s like a chain reaction for me which is very beneficial…* (P9, non-ECR, LMIC)

PhD students undertaking distance-learning PhDs at the University of Bristol had supervisory teams comprising local and United Kingdom supervisors. ECRs reported no tensions balancing joint supervision. Relationships with supervisors were described in positive terms such as *supportive, caring* and *hands-on*. One limitation to PhD supervision by United Kingdom researchers was that it was mostly conducted remotely. Time zones and Internet connectivity did not present major barriers, but one participant felt that relationships with their supervisors really developed during face-to-face working during annual workshop weeks and study visits to the United Kingdom.

*Nothing beats interacting with someone in person, although we have this advantage of this technology. So yes, sometimes I feel that if only I was not a distance learning student and I could meet them often.* (P8, ECR, LMIC)

Some United Kingdom participants discussed a lack of distinction between their roles as mentors and supervisors. They felt that there were sometimes too many people involved in supervision arrangements with unclear roles and boundaries. This was not, however, reflected in comments from ECRs.

#### Training and other opportunities

ECRs discussed the training and other development opportunities that were made available to them. Some ECRs attended short courses in the United Kingdom, covering topics such as gender-based violence and research skills such as qualitative and mixed-methods research which may not be available in their home country. The opportunity to attend international training was highly valued; it also provided important networking opportunities. One distance-learning PhD student discussed the limitation that much of the training offered by their registered United Kingdom university was in person. COVID-19-related lockdowns presented an unanticipated benefit as more training moved online. Many ECRs participated in online training courses. Some ECRs took part in face-to-face or online research methods training with United Kingdom mentors, which was very well received.

Most ECRs had attended one or both of the annual HERA international workshops and were very positive about this experience. The agenda was developed through a group prioritization exercise; training was delivered on the most popular research methods. Several participants enjoyed the *practical*, *hands-on nature* of training sessions and group discussions. Participants were also able to learn from the findings and experience of other country teams. There was increased participation of ECRs in the second annual workshop, with a separate ECR session which participants valued as a space for open and honest sharing and discussion about career development. Senior researchers were encouraged to present jointly with ECRs who described this as a great opportunity.

*…that’s another opportunity you know because I get a bit anxious of course to talk to like these amazing people who are big researchers and we read all their papers and they’re so cool you know and to talk about our research and our findings…* (P13, ECR, LMIC)

Opportunities created for ECRs by country PIs included participation in training, including English language training, attendance at meetings or conferences and delivery of presentations or training. Other opportunities afforded by engagement with the wider international HERA team included participation in meetings, delivering teaching, taking on leadership roles, collaborating on writing a funding proposal and developing international networks.

*I have attended quite a lot of short courses, which is part of my PhD funding as well. Also apart from that the HERA project also gives me opportunities to attend like for example if [principal investigator] or somebody has meetings or workshops, trainings, we’re allowed to go and attend that as well, whatever conferences we can attend so it has given a lot of opportunities for those sort of activities that I otherwise would not get involved in.* (P5, ECR, LMIC)

Inequity of RCS opportunities through HERA was created by several factors. Communication of opportunities was felt by one ECR to be inconsistent, leading to missed opportunities. The structure of funding also created inequity between ECRs. Three ECRs in the group were undertaking distance-learning PhDs at one of the lead United Kingdom universities. The funding body allocated these doctoral students a separate budget for research training and travel, and they were considered to have *more benefits* than other team members. In addition, RCS opportunities provided centrally by the funder were often only accessible to these PhD students. This presented an issue when a group of ECRs wanted to collaborate on planning an academic English writing workshop, but only a centrally funded PhD student was allowed to submit the grant proposal for a workshop.

*…because we don’t have any ECRs funded by the group, we don’t have the same opportunities, but I don’t think it has—like I don’t think this is something that has to do with the HERA team, it has to do with the funder.* (P2, ECR, LMIC)

#### Funding and resources

ECRs from LMICs were generally positive about their experience of working with an international funder and found it comparable to previous experience with other national or international funders. Whilst there were some administrative and reporting requirements, these were not regarded as burdensome, and some described them as simple. United Kingdom participants were more critical about the requirements of the funder and expressed concern at the processes required, including the requirement for funds to be distributed via the lead United Kingdom institution; this was perceived by one participant as *neocolonial* and a *replication of control* (P6, non-ECR United Kingdom). Several participants reported problematic delays in payment to partner countries or PhD students, especially early on in the project.

Beyond providing training opportunities as discussed, participants reported that funding enabled provision of three important factors: *salaries, infrastructure* and *journal access*. Salary provision was important, especially where there was limited local funding available for research salaries in academia. One participant described how the salary provided by HERA was significantly higher than local academic salaries, enabling them to step away from clinical work to focus on research. Strengthening of research infrastructure included the purchasing of computers, software and office supplies. Participants felt that there was good provision of these resources and that the impact would persist after the funding ended. One participant explained that provision of these resources helped validate their role as researcher:

*I think those things that seem really small make a difference because it gives the impression the academic work is actual work where it’s not something that everyone understands and not something that you can just do wherever you are.* (P2, ECR, LMIC)

Several ECRs valued the access to online libraries and journal articles provided by the project. This enabled engagement with academic literature that they would not otherwise have been able to access and was seen as more efficient than previous portals used to access academic papers.

### Informal or unplanned elements of the RCS programme

#### English language

In our group, English language ability varied significantly between LMIC ECR researchers; some spoke English as their first language; some were fluent and had completed English language education; others had limited written and spoken English. The impact of English language ability on the ECR experience of RCS was described in two subthemes.

- *Engagement with the international group*
  ECRs with limited English language ability experienced barriers to participating in training, as well as attending meetings and workshops with the wider international HERA group. This barrier was highlighted by only one of the United Kingdom team members, but identified by several LMIC ECRs. LMIC group members advocated for the funding of English language training, which was approved and facilitated by country PIs.
  *We discussed how this is a major challenge to advance careers in low and middle income countries that don’t speak English as their native language then maybe we should get some money to get English lessons for them then we can improve the work a lot within the global health group.* (P13, ECR, LMIC)
  Whilst the necessity to communicate with the wider group in English presented a challenge, it was also seen by some as a learning opportunity.
- *English language academic writing required for international publication*
  LMIC group members highlighted how limited written English language ability acts as a barrier to publication in international journals. Even those with advanced English language ability reported that they sometimes required United Kingdom mentors or supervisors to correct their written English. In response to this development need, several ECRs worked collaboratively with the capacity-strengthening lead to develop a successful funding proposal for an English language academic writing workshop for ECRs which has since been conducted online.
  One ECR participant discussed the way in which international academic publishing not only requires the use of English language, but also incentivizes the use of academic theory from Global North countries. English language in academia was perceived as more than just a language, as international publishing requires *adjusting your thoughts to the thinking of those places*. Another participant reported how LMIC researchers are less likely to get published unless collaborating with HIC researchers.
  *We actually do have experiences of just receiving no’s when we are first authors in international journals alone, you know, and we don’t have [any] one from the United States or Europe together in the authorship, that’s a bit harder, it’s less value I guess the work that we do produce here.* (P13, ECR, LMIC)

#### Open relationships and communication

One of the most dominant themes to emerge from the interviews was the open nature of relationships and communication within HERA. Relationships were described as *open*, *friendly*, *informal*, *nonhierarchical*, *supportive*, *comfortable*, *approachable* and *relaxed.* The development of personal relationships was strengthened during face-to-face meetings, especially during the annual workshops. These convivial relationships carried over into remote working and even online friendships. Some ECRs described how HERA leaders and senior team members set the tone for these nonhierarchical relationships and proactively engaged with ECRs.

*P: …actually, the first day we didn’t talk and we didn’t much try to talk to them, but at the final day we were like friends, and they facilitate us to behave like that I think.*

*I: That’s interesting, what do you mean by that, how do they facilitate that?*

*P: Actually, every time they tried to talk with us and they tried to discuss with our project and our current involvement, about our barriers and also about our academic things, they always tried to talk with us. They did not allow us to be alone, they always tried to get our involvement in a workshop.* (P10, ECR, LMIC)

Some ECRs reflected on how these nonhierarchical relationships contrasted with more traditional hierarchical relationships in their home academic environment, although one ECR positively described the non-authoritative yet clear hierarchical structure in their home country team. The nonhierarchical nature of the HERA group enabled ECRs to join discussions, share ideas and give feedback in ways that some were not accustomed to. This open communication acted as an RCS facilitator as it encouraged participants at different levels of seniority to share their ideas, which facilitated their learning.

*You don’t have the hierarchy sort of thing, and you don’t feel scared to actually go ahead and give an idea or talk about something, so I think that’s really important when you’re trying to learn and build skills so that you don’t feel stifled.* (P5, ECR, LMIC)

#### Connection and disconnection

ECR participants discussed the varying degrees to which they felt connected to or disconnected from their national teams and the international group. One of the priorities of the RCS programme was to engage ECRs across the different country teams and to facilitate the development of an ECR network. Face-to-face meetings such as the annual workshops were important ways of building relationships, creating connections and a sense of ease between ECRs in different country teams. Bimonthly online ECR peer support and education meetings were established and facilitated by the capacity-strengthening lead. Those involved in the meetings valued the sense of connection and mutual support provided; this was especially important for researchers working remotely or in isolation. Some maintained contact via email or WhatsApp to discuss their ongoing work, learn from each other and share resources.

*We do have the early career group researchers meeting every 2 months, and it’s a big support group to talk about our anxieties of course of starting our careers and things like that but also to discuss some very crucial and important things and next steps.* (P13, ECR, LMIC)

Remote working with colleagues from other countries posed a barrier to connection, but regular virtual meetings and staying in contact by email, WhatsApp and social media helped to lessen this disconnect to some degree. Most participants reported that Internet connectivity was not a major issue. Time zone differences were a challenge for some when participating in international meetings.

*What’s frustrating for me being a PhD student and sometimes distance learning, it sometimes gets lonely and quite frustrating sometimes because, I mean we do have the early career researchers group, but I think everyone being so far away makes it very impersonal, and also the time difference and everybody’s busy with their own thing, so I mean if there is anything that’s a minus, that’s what I’d say is quite difficult.* (P5, ECR, LMIC)

Physical distance and geopolitical barriers created disconnection for those not colocated with their country team. On a practical level, this limited the ability to meet in person or work in a shared office space. Lockdowns imposed due to COVID-19 enforced remote working for colleagues who had previously worked together, thus limiting opportunities for informal interaction. A number of ECRs had to balance their research activities alongside other professional commitments, including clinical practice. Competing work pressures limited the time they were able to commit to research, meet with colleagues and avail of opportunities presented by the international team.

*It’s very problematic because we wanted to meet for the very long time, but the problem is we can’t arrange a time which meets the demands and the needs of every member because we are six, and every one of us has their own obligations and own jobs, and so it’s quite problematic.* (P16, ECR, LMIC)

#### Diversity

Diversity manifested in varied ways within the group. Within and between the country teams, there was a range in research experience as well as methodological knowledge and skills. This enabled the sharing of knowledge and teaching at a group level, although one senior researcher reflected on this as a difficulty when planning training to meet the needs of a diverse group. The different research teams were working in diverse academic and institutional contexts, with different academic processes, practices and infrastructure, which needed to be recognized.

*…in a group like HERA where we have so many different cultures, and academia is so different within each culture, that we learn to become almost like better human beings, or more respectful of our academic differences and backgrounds.* (P18, non-ECR, United Kingdom)

Many participants valued the *diversity* of *perspective* that came from working with mentors or supervisors from different countries. Sometimes their own interpretation of data was challenged, new ideas emerged and they were pushed to think *outside the local level*. Different perspectives also came from sharing of experience with the other country teams in HERA, although international evidence and policies needed to be adapted to the local level, and solutions from one country did not always translate well to another context.

*So, it's always interesting to add a new person to the group because you always get a new perspective, and sometimes it might be conflicted to your own and you would… I would be like “oh, maybe it is like this”, you know, so it's a new perspective and a new knowledge exchanged that is happening when you have a separate person involved.* (P7, ECR, LMIC)

### Sustainability of RCS

Participants discussed the sustainability of the RCS programme at the level of the individual researcher, research team and research network. This is summarized in Table 3 below.

**Table 3:** Sustaining RCS at different levels

Individual	• Securing future research funding for individuals • Career progression
Country	• Developing expertise as a country team • Readiness to continue research independently • Spread learning locally
Research network	• Early stakeholder engagement • Developing a network of experts • Establishing connections between country teams • Future opportunities for research collaboration

(i) *Individual researcher*
  ECRs may face the challenge of securing further funding to pursue academic work after HERA. Local funding streams are not always accessible, and funding opportunities can be particularly limited for mid-career researchers. Career progression of ECRs was considered one measure of successful RCS, which could be supported through development of CVs [curricula vitae], facilitating publication and supporting individual applications for further funding.
  *In terms of capacity […] can I see trajectories of team members moving from their initial kind of induction into the team to seeing them, you know, deliver particular bits of data and analysis, are they then co-authors on papers, are they then applying to do a Master’s or a PhD.* (P15, non-ECR, United Kingdom)
(ii) *Research team*
  Participants felt that their strengthened research teams were developing an expertise within their region. The degree of readiness to continue this work varied between the country teams, with some requiring ongoing input, whilst others aspired to become regional leaders in the field. Several participants discussed ways in which the learning that occurred during the project could continue to be spread locally by a *chain reaction, ripple effect* or *roll out of knowledge.* However, one participant highlighted that to sustain this process requires planning and also investment, suggesting a *train-the-trainer* model.
(iii) *Research network*
  Early engagement with local stakeholders, including community and government officials, was considered important in securing their ongoing support for the work.
  *Regarding the stakeholders' formation, I think this has created a road to sustainability because we want the local government as well as the people in charge of the [clinics] where we are working to take ownership of the activities that we will be doing later on, so if someone is taking the ownership, then even after the research is completed, then this can definitely lead to sustainability factor.* (P7, ECR, LMIC)
  The *network of experts* within HERA would continue to be a source of knowledge and guidance for ECRs even once the project concludes. HERA has provided a platform to establish connections not only between partner countries, but also with institutions outside of the group as part of an international network of violence against women researchers; this may open opportunities for future joint funding applications and research collaboration.

## Discussion

Our interview data highlighted important planned and unplanned elements related to the ECR experience of our RCS programme. We contextualize our findings within the existing literature, highlight adaptations made to our programme in light of study findings and conclude with considering future directions and sustainability.

### Planned elements

Our interviews explored some of the planned elements of our RCS programme, which have also been highlighted as important in existing literature. Positive mentoring and supervision relationships are a key influence on ECRs, and the supervisor or academic adviser may impact academic success or career progression [8, 11]. Existing studies highlight potential issues, including lack of access to good-quality supervision [18, 23], inconsistent quality and frequency of supervision [11] or mismatched expectations about the role of supervision [11, 16]. Our ECR participants were very positive about their relationships with their supervisors and reported frequent or adequate contact, whether in person or remotely. Other studies have reported challenges in joint supervisory arrangements, with tension balancing time spent on doctoral studies versus contributing to the wider project [16]. Our doctoral students reported good relationships between their United Kingdom and home country supervisors; this may reflect collaborative working relationships within the wider HERA research project. Role confusion can be an issue when there are multiple senior people involved in supporting ECRs from different disciplines; the lack of clarity between mentor and supervisor was highlighted by some United Kingdom non-ECR participants. As participation in the interviews by LMIC senior researchers was limited, we are unable to reflect on their experience of supervision relationships.

Participants appreciated the training and other opportunities provided through HERA. International short courses were particularly valued for high-quality training as well as providing important networking opportunities. Participants did not describe any courses as inadequate or failing to meet their training needs, as reported in other studies [11]. The workshop weeks were particularly valued for offering highly relevant participatory training in research methods, valuable presentation experience and networking opportunities. The opportunities created by working on HERA which went beyond formal training, such as gaining teaching or leadership experience, are difficult to formally plan in an RCS programme but were generated by productive relationships with supervisors and colleagues in the wider group. Criteria for accessing development opportunities directly through the funder created inequity in opportunity, which contradicted one of our core values and was fed back to the funder. We also advocated for funded PhDs to be available at LMIC institutions, and the funder now fully funds LMIC students wishing to register for a PhD at a university in their home country as well as other formal qualifications such as bachelor’s and master’s degrees.

Through evaluating our RCS programme, we have recognized the importance of engaging LMIC group members in developing the RCS agenda. We since appointed two LMIC ECRs as joint-training leads alongside the United Kingdom capacity-strengthening lead. It is hoped that they will be able to better represent the training needs of LMIC group members and enhance the sense of shared ownership of the agenda. Our funder also now encourages the appointment of LMIC training leads in their global health research groups.

Funding is critical to enable ECRs to engage in research; existing literature highlights that lack of access to reliable or sustainable funding is often an issue in LMICs [13, 17, 18]. As reported elsewhere [10, 16], many of our ECRs were juggling their academic careers alongside other personal or professional commitments. The viable salary provided by the project offered some economic security and enabled protected time for academic work. Sustainability of funding is a critical issue in LMIC global health research [3], and in order to maintain the impact of RCS, global health funders should consider committing to medium or longer-term funding. The experience of working with the funder was generally positive. However, problematic processes identified included allocation of funds through the United Kingdom university, payment in arrears and reduced percentage lead-time funding for preparatory work on the project. Delays in payment to LMIC academics may result in them working for free or incurring a personal cost to pay soft-funded researchers on their team. This could be considered a practical manifestation of global power imbalances. The project administrator advocated for a change in payment processes at the grant-holding United Kingdom university to enable payment to PhD students and partner institutions in advance rather than arrears; this is an example of identifying and modifying a structural barrier. Funders must recognize that research projects typically require up-front funding to cover research costs. They must also seek to understand the financial realities of staff in different contexts and adapt funding processes accordingly so that individuals are not required to work without payment for prolonged periods whilst awaiting the release of funds. Increasingly, some global funding streams now authorize LMIC partners with good research infrastructure to host the grant. Capacity-strengthening programmes should support the development of administrative and financial capacity within collaborating research institutions with a view to enabling future grant-holding independence.

### Unplanned elements of our capacity-strengthening approach

Some important unplanned elements of our RCS programme emerged from the interviews. English language has been normalized as the dominant academic language in global health research; this has often gone unquestioned. Research is now exploring how this may present a barrier to advancement within global health research and practical solutions to mitigate this [24]. Participants identified academic English language ability as a barrier to participation in the RCS programme, and indeed to engagement with the wider international research group; we responded with the funding of English language training. At the wider structural level, funding calls promoted in English and English-language-only application processes [18, 20] can act as a barrier to LMIC researchers successfully obtaining funding. Other practical mitigating measures that could be considered in future programmes include providing translators to enable participation in important meetings or training, translating important group emails and offering English language support for the completion of funding applications.

Our participants felt that English language ability could act as a barrier to international publication in global health. Supporting ECRs with academic writing and navigating the process of publication in international journals was recognized as an important development need, and HERA supported a group of ECRs to win funding for and deliver a successful academic writing workshop. The pressures to publish in international journals incentivizes LMIC global health researchers to publish in English, alongside English-speaking authors and using academic theory originating from the Global North. Publication for the international audience may mean that the impact of some research is lost for those working at the local or regional level [25]. Indeed, the Brazilian HERA team plans to publish some of their findings in Portuguese in national journals where it may have more impact on local researchers and practitioners.

One of the strongest themes represented in the data was that of open relationships and communication. Some ECRs reflected on how these relationships differed from more formal or hierarchical relationships that they were used to. The positive nature of the relationships enabled both productive supervision and mentoring, but also emboldened ECRs to engage in meetings and trainings and therefore facilitated some of the planned elements of the programme.

ECR interview participants described experiences of connection and disconnection that either enabled or acted as barriers to engagement in the RCS programme. The importance of connection and network development is recognized in the existing literature [17]. Some participants highlighted the value of face-to-face meetings to strengthen relationships, which were then maintained remotely [26]. Some LMIC researchers may be at particular risk of scientific isolation from professional peers [27], and a remote support networks, such as our online peer support group, can go some way to mitigate this. Some of the causes of disconnection can be modified, for example, provision of English language training and ensuring careful consideration of time zone differences for international meetings. Productive connections forged between ECRs may lead to future research collaborations as ECRs develop into academic leaders of the future. Subsequent to these interviews, a group of HERA ECRs collaborated with another global research group to develop and run a 3-day online workshop with interactive lectures covering topics including pursuing a career in global health and female leadership. This workshop provided an opportunity to develop leadership skills and networks with other international researchers.

The diversity of group members’ research skills, disciplines, experience and perspective was mostly considered beneficial and led to a rich research environment. This cannot be completely planned, but is one of the many benefits from working in partnership with academics in different countries and contexts. Offering new or alternative perspectives on research design and data analysis can be fruitful, with learning and reflection happening in all directions between country teams. In the sensitive field of gender-based violence (GBV) research, findings must be interpreted within the sociocultural context, and the research agenda retained under local ownership.

### Strengths and limitations

Our study has several strengths and also some limitations. RCS programmes are often complex, and outputs can be difficult to define and measure; our qualitative study offers a richer understanding of the experience of engaging with RCS. Our study gives voice to and prioritizes the experience of ECRs, who can be underrepresented in the literature, despite often being the main recipients of RCS efforts.

We had a moderate level of participation from the group with all countries, genders and level of seniority represented by interview participants. There was very limited participation from middle- and senior-career researchers from the LMIC teams. Although male and female researchers participated in the interview, we did not explore their perceptions of how gender impacted on their experience; this was a missed opportunity. We had minimal participation from researchers with limited English language ability, and no one utilized the option to be interviewed using a translator. The perspective on academic English language ability therefore comes indirectly from English-speaking team members.

We attempted to introduce independent objectivity by engaging two external research assistants to conduct the interviews and conduct data analysis, but the lead researcher was the United Kingdom-based capacity-strengthening lead, and the study supervisor was the United Kingdom-based co-PI. This may have led to selection bias with group members opting out of interviews, or response bias with participants giving desirable answers. However, our closeness to the programme may have enabled a deeper understanding of the data and themes that emerged. Capacity-strengthening has been a core focus in our group with key concepts explored in workshops; this may have influenced participant responses. We maintained confidentiality within the research team by ensuring that the study supervisor only viewed anonymized extracts, not the raw data.

When conducting qualitative research, it is important to practice reflexivity to understand how our own positionality influences data interpretation [28]. The three main researchers who collected data and undertook the majority of data analysis were all United Kingdom-based ECRs. Whilst we had empathy with the career stage of ECR participants, our United Kingdom positionality means that we may have misunderstood aspects of the experience of LMIC researchers. A senior Brazilian researcher joined the study team later on, bringing a valuable LMIC perspective and highlighting certain pertinent issues in the data and its interpretation. Our shared team approach to coding and thematic analysis helped to reduce individual subjectivity when interpreting the data.

### Future directions and research

As this study forms part of an ongoing process of M&E, our findings were fed back to the group and led to real-time adaptations of our RCS programme. Future RCS priorities must be embedded with a sustainability focus and include the following:

- Further analysis of this interview data to explore different research questions and publish with improved LMIC authorship representation.
- Facilitating opportunities for ECR authorship on papers submitted by our group to international academic journals.
- Supporting ECR career development, particularly the transition to doctoral and post-doctoral work.
- Expand appropriate RCS support to middle- and senior-career researchers.
- Support applications for future funding, thereby providing stability for individual researchers and research teams, and sustainability for research projects.
- In order to sustain the impact of RCS programmes, RCS should be embedded as a research theme in future funding applications with funding allocated for M&E as well as strategies for creating lasting impact, such as investing in a train-the-trainer model.
- Move towards decentralized ownership of RCS activities. One example of learning, which has been embedded in a sustainable way, is the integration of gender-based violence into the curriculum for a master’s degree in nursing and public health at one of the partner universities. HERA has also supported the development of locally delivered qualitative research methods training courses in two of the partner countries.
- Support the development of research partners as regional experts/hubs in gender-based violence and health, including development of the skills and infrastructure required to lead future global health research programmes.

This research study and subsequent group conversations have highlighted fruitful areas for further research within our group. How do issues of power and partnership manifest in a global health research group and specifically within an RCS programme? Does RCS have the potential to address some of the power imbalances? How to manage the practical barriers and inequity introduced by English language predominance in a global health group working, including how to engage researchers with limited English language skills in comparative or synthesis work where bilingual data analysis may be required. How to support ECRs from LMICs to be successful in both local and international academic publication, whilst challenging some of the structural barriers in international global health publishing?

## Conclusions

Our study set out to provide an in-depth qualitative exploration of the ECR experience of engaging with an RCS programme. Exploring ideas with participants enabled us to identify ways in which both planned and unplanned elements of our RCS programme can serve as facilitators and/or barriers to engaging with RCS; other global health groups may also find the identified themes pertinent to consider. As this study formed part of an ongoing evaluation, we were able to make real-time adaptations to our RCS programme to try and better meet the needs of our ECRs and respond to barriers identified. At the group level, we identified some practical issues leading to inequity and were able to advocate for process change within our institutions and with the funders. Some of the findings from our interviews highlight challenges at the structural level of global health funding and international publication, which are being increasingly discussed in the literature. These issues feed into important ongoing debates about structural power in global health, how to make it a more equitable space and the role of RCS in addressing these power imbalances.

## Data Availability

The datasets generated and analysed during the current study are not publicly available due to maintaining the confidentiality of individual participants. They will be stored at the University of Bristol Research Data Storage Facility, and anonymized data may be available on request by academic researchers.

## Abbreviations

HERA: HEalthcare Responding to violence and Abuse
RCS: Research capacity-strengthening
M&E: Monitoring and evaluation
ECR: Early career researcher
LMIC: Low- and middle-income country
HIC: High-income country
PI: Principal investigator
